# Current Approaches for Personalized Therapy of Soft Tissue Sarcomas

**DOI:** 10.1155/2020/6716742

**Published:** 2020-03-31

**Authors:** Kirill I. Kirsanov, Ekaterina A. Lesovaya, Timur I. Fetisov, Beniamin Yu Bokhyan, Gennady A. Belitsky, Marianna G. Yakubovskaya

**Affiliations:** ^1^N. Blokhin Cancer Research Center, Moscow, Russia; ^2^RUDN University, Moscow, Russia; ^3^I. P. Pavlov Ryazan State Medical University, Ryazan, Russia

## Abstract

Soft tissue sarcomas (STS) are a highly heterogeneous group of cancers of mesenchymal origin with diverse morphologies and clinical behaviors. While surgical resection is the standard treatment for primary STS, advanced and metastatic STS patients are not eligible for surgery. Systemic treatments, including standard chemotherapy and newer chemical agents, still play the most relevant role in the management of the disease. Discovery of specific genetic alterations in distinct STS subtypes allowed better understanding of mechanisms driving their pathogenesis and treatment optimization. This review focuses on the available targeted drugs or drug combinations based on genetic aberration involved in STS development including chromosomal translocations, oncogenic mutations, gene amplifications, and their perspectives in STS treatment. Furthermore, in this review, we discuss the possible use of chemotherapy sensitivity and resistance assays (CSRA) for the adjustment of treatment for individual patients. In summary, current trends in personalized management of advanced and metastatic STS are based on combination of both genetic testing and CSRA.

## 1. Introduction

Soft tissue sarcomas (STS) present a highly heterogeneous cancer group with more than 50 subtypes in terms of anatomical location, histology, molecular characteristics, and prognosis [1]. STS originate from mesenchymal cells of a number of tissue lineages, such as adipose, muscle, fibrous, cartilage, and vasculature [2]. Surgery is the standard of care for primary soft tissue sarcomas, while for locally advanced or metastatic STS, chemotherapy is generally the principal treatment modality [3]. Factors to consider when selecting first-line chemotherapy for advanced STS include, besides histological state, disease bulk, and patient-related factors such as age and presence of comorbidities, genetic and molecular-biological peculiarities of specific tumors. Recent discoveries of the underlying molecular and genomic mechanisms of different STS histology subtypes have enabled to reclassify these tumors and to optimize cytotoxic chemotherapeutic regimens as well as to encourage development of novel targeted chemotherapeutic agents in treating STS. Thus, the development of low molecular weight inhibitors of chimeric kinase ETV6-NTRK3, specific for congenital fibrosarcoma/mesoblastic nephroma, is now at the Phase I clinical trial ([4] and Table 1). Efficacy of tyrosine kinase inhibitors imatinib and sunitinib was approved for COL1A1-PDGFB-positive dermatofibrosarcoma treatment or involved in clinical trials of Phase I-III ([5–7] and Table 1). The first part of this review will summarize the approaches of targeted STS therapy based on genetic alteration associated with distinct tumor types. However, the prognosis of personalized chemosensitivity and resistance of STS presents certain difficulties, as accumulated data are insufficient to provide the efficacy of prescribed therapy of at least 40% or more. Moreover, less than 40% of STS subtypes could be characterized by specific chromosomal translocation, oncogenic mutation, or gene amplification. The adjustment of effective therapy strategy for STS forms without any molecular-genetic peculiarity remains a lottery game with unpredictable outcome. Phenotypic or functional screening can be an alternative to overcome this gap. It refers to the identification of antisarcoma activity of individual drugs or drug combinations using cell- or tissue-based models: chemosensitivity and resistance assays (CSRA). The development of CSRA was started in 1970s for identification of anticancer drugs for individual patients, and the first assays were based on colony-forming efficiency of tumor-derived cells in the presence of various drugs [8, 9]. Furthermore, CSRA were incorporated in a variety of detection systems but shared similar principles and general procedures: (a) tumor cells were isolated and established in an *in vitro* medium; (b) cells were incubated with the chemotherapeutic agent(s) of interest; (c) cell survival (or death) was assessed; and (d) a report detailing sensitivity and/or resistance to tested drugs was generated. Now, CSRA and patient-derived xenografts (PDX) are considered as an efficient approach to identify treatments or new therapeutic indications for approved drugs [10, 11]. In the second part of the review, we will discuss possible use of CSRA for the optimization of sarcoma treatment and current progress in the field.

## 2. Molecular Targeting Therapies for STS

### 2.1. Inhibition of Target Oncogenic Protein Expression or Activity

Design and development of small molecule inhibitors have resulted in remarkable progress for treatment of certain cancers, particularly with drugs targeting protein kinases. Direct inhibitors are expected to work by specific binding and promotion of degradation and/or by specific binding and blocking sites required for target protein activation or interaction with other critical effector proteins. Certain progress has been made in directly targeting many transcription factors, in particular, chimeric kinases and mutant proteins involved in cancer development ([12] and Tables 1 and 2). However, the design of direct inhibitors for wild-type and fusion transcription factors can be attributed in part to the large protein-protein interaction interfaces and absence of deep protein pockets that are common targetable sites for drug design [13, 14]. Only a few molecules were described, designed as inhibitors of STS-specific aberrant proteins, which reached clinical trial. For example, LOXO10, an experimental, highly selective inhibitor of tropomysin-related kinases (TRK), is now involved in clinical trials of Phase I for treatment of infantile fibrosarcoma [4], which is nearly always characterized by a t(12; 15) (p13; q25) translocation [15]. This translocation fuses the ETS variant gene 6 (ETV6) in chromosome 12 with the neurotrophin 3 receptor gene (NTRK3) kinase domain, resulting in activation of multiple signaling cascades including the RAS and PI3K-AKT pathways [16]. Another small molecule, YK-4-279, is able to disrupt binding between fusion protein EWS-FLI1, specific for Ewing's sarcoma [17], and RNA Helicase A (RHA), which is necessary to enhance the oncogenic activity of EWS-FLI1 [18]. Therapeutic efficacy of YK-4-279 was proved in *in vitro* and *in vivo* models [19]. The YK4-279 analog TK216 is currently being used in a Phase I clinical trial in patients with relapsed or refractory Ewing's sarcoma [20]. Anaplastic lymphoma kinase (ALK) is a receptor tyrosine kinase involved in the genesis of several human cancers, in particular, inflammatory myofibroblastic tumor (IMT), which is characterized by ALK-based chromosomal translocations t(2; 19) (p23; p13.1), t(1; 2) (q22-23; p23), t(2; 17) (p23; q23), t(2; 2) (p23; q23), etc. [21]. To date, FDA-approved ALK inhibitor Crizotinib is undergoing clinical trials of Phase II-III for IMT treatment ([22] and clinical trial NCT03874273), and several small molecules with the potency to inhibit ALK are under investigation [23]. Liposarcomas and intimal sarcomas form distinct STS subset, where MDM2 and CDK4 gene amplifications serve as oncogenic drivers as well as therapeutic targets [24]. Low molecular weight inhibitors were described for both genes, in particular, CDK4 inhibitor palbociclib is currently involved in clinical trials [25], and experimental MDM2 inhibitor demonstrated anticancer activity *in vitro* and *in vivo* [26].

Genetic inhibition of fusion gene by antisense oligonucleotides or siRNA could be another option. Thus, it was reported that antisense oligonucleotides and siRNA inhibited expression of EWS-FLI1, chimeric gene specific for Ewing's sarcoma, in cell cultures and in the xenograft model *in vivo* [27], as well as decrease of SS18-SSX1 gene expression in synovial sarcoma [28].

As modeling and designing of direct inhibitors for known fusion genes/proteins are challenging, other strategies have been explored. There has been significant progress in several approaches, such as targeting transcriptional coactivators, phosphorylation modulators, and upstream regulators of chimeric kinases. Phosphorylation of EWS-FLI1 could be disrupted by EngerlinA, an active constituent of the plant *Phyllanthus engleri*, as it was demonstrated *in vitro* [29]. Furthermore, epigenetic regulators histone deacetylases (HDACs) and demethylases (DMT) and DNA repair enzyme poly-(ADP-ribose) polymerase 1 (PARP1) are transcriptional partners of EWS-FLI1, which formed a solid ground for use of PARP inhibitor olaparib, HDAC inhibitor entinostat, and DMT inhibitor HCI-2509 in preclinical and clinical studies for Ewing's sarcoma therapy [27, 30, 31]. Efforts have been made to find the kinases, which are responsive for PAX3-FOXO1 phosphorylation, and to attenuate their activity using siRNA or known inhibitors [32]. Moreover, epigenetic regulators influence PAX3-FOXO1 interaction with transcriptional partners as well. Therefore, use of HDAC inhibitors in the treatment of PAX3-FOXO1-positive rhabdomyosarcoma represents attractive therapeutic strategy [32].

Known FDA-approved low molecular weight inhibitors, such as already mentioned PARP and HDAC inhibitors, form a separate group of targeted drugs for STS treatment. Drug repurposing refers to the application of a drug for another indication than it was originally approved for. It has received increasing interest as an alternative strategy to de novo drug synthesis [33] as usage of known therapeutics gone through preclinical and clinical studies could drastically decrease the time and the cost of investigations. Moreover, it meets the demand of social changes from overconsumption to rational reuse and recycle. Despite the main problem in the drug repurposing approach associated with inability to patent the known drugs for novel application and, therefore, with certain difficulties in fundraising and absence of massive studies, there are a number of repurposed medical, in particular, in cancer treatment. There are different approaches to drug repurposing including target-based, integrating disease-associated proteins, biomarkers, and pathway knowledge to identify a specific new target or mechanism and therapeutic use; drug-based, identifying similarities between molecular structures of existing drugs; and disease-based, finding new strategies to drug intervention in a disease. Significant example of the identification of a new target is imatinib mesylate, initially designed as a BCR-ABL inhibitor and used in patients with chronic myeloid leukemia (CML). Imatinib was found to specifically target PDGFRB tyrosine kinase, and so its use was approved for treating COL1A1-PDGFRB-positive dermatofibrosarcoma [5]. Based on the same strategy, tyrosine kinase inhibitor sunitinib was included in clinical trials for imatinib-resistant dermatofibrosarcoma therapy [6, 7]. Furthermore, the drug repurposing approach is even more applicable for STS with oncogenic mutations: gastrointestinal stromal tumors (GIST) with activating mutations in c-KIT, PDGFA and BRAF, myxoid round cell liposarcoma with activating mutation in PI3K/Akt signaling component PI3CA ([24] and Table 2). Multitargeted low molecular weight inhibitors as imatinib, nilotinib, pazopanib, sorafenib, sunitinib, dabrafenib, vemurafenib, mTOR, and PI3K/Akt inhibitors have demonstrated their anticancer activity and therapeutic potential for treatment of STS in preclinical and clinical studies [34].

Special attention should be given for marine-derived natural product trabectedin, initially isolated from the marine ascidian Ecteinascidia turbinate [35]. Currently, trabectedin is a validated option for the treatment of patients with advanced STS as target molecule [36]. The compound was described to interfere directly with activated transcription, to poison the nucleotide excision repair system, and to generate double-strand DNA breaks (DSBs) [36]. The drug has shown a high selectivity for myxoid liposarcoma, characterized by the translocation t(12; 16) (q13; p11) leading to the expression of FUS-DDIT3 fusion gene. Trabectedin appears to bind directly with the chimeric protein and to impair transactivating activity of FUS-CHOP [37]. Similar results were obtained for trabectedin and EWS-WTI1-positive desmoplastic small round cell tumor [38], myxoid liposarcoma with EWS-DDIT3 [39], and Ewing's sarcoma with EWS-FLI1 translocation [40].

### 2.2. Inhibition of Downstream Effectors of Oncogenic Protein

All oncogenic genetic alterations in STS start aberrant activation of transcription through multiple downstream targets whose expression is proposed to promote tumorigenesis. High-throughput technologies such as DNA microarray, RNA sequencing, and chromatin immunoprecipitation sequencing (ChIP-Seq) have enabled generation of comprehensive signatures of downstream targets expressed in specific STS subtypes driven by oncogenic mutation or chromosomal translocation. Moreover, this approach is also applicable for sarcomas with complex karyotypes, which often lack tumour-specific genetic abnormalities.

The insulin-like growth factor (IGF) signaling contributes to tumorigenesis through IGF1R phosphorylation and activation of several cancer-related pathways to regulate cell growth and tumorigenesis in a variety of malignancies [41]. It is known that EWS-FLI1 protein, which is characteristic for Ewing's sarcoma, binds the promoter of insulin-like growth factor binding protein 3 (IGFBP3) to suppress the expression of IGFBP3, which sequesters circulating IGF1. These results suggest a crosstalk between the oncogenic function of EWS-FLI1 and the IGF1R signaling [27]. Similar observation was made for EWS-WT1-positive desmoplastic small round cell tumor [42]. Based on these findings, the studies of anticancer activity of IGF1R inhibitors, monoclonal antibodies R1507, MK-0646, cixutumumab, ganitumab, and figitumumab were started for several STS subtypes; some of them reached clinical trials [27, 42].

Endoglin, coreceptor of the TGF*β* family, is an interesting and perspective target for Ewing' sarcoma as its high expression is associated with poor prognosis of the disease. Thus, monoclonal endoglin-targeting antibodies, TRC105, OMTX503 and OMTX703, have demonstrated the decrease in tumor growth in Ewing sarcoma cell line-derived xenografts and patient-derived xenografts as well as in angiosarcoma in clinical trials of Phase I/II [43, 44].

It was reported that EWS-FLI1 protein upregulated the expression of Aurora kinases A and B (AURKA, AURKB) by direct binding to their promoters [45]. As AURKA and AURKB are serine/threonine kinases, regulators of mitosis, and diverse signal transduction pathways, and their overexpression is associated with tumorigenesis, they have become a promising therapeutic option in cancer therapy [46]. Efficacy of antitumor action of AURK inhibitors MLN8237 and tozasertib was demonstrated *in vitro* on Ewing's sarcoma cell lines and *in vivo* in xenograft models [27].

Ezrin, an ezrin-radixin-moesin protein, presents another interesting target for STS therapy. This protein links the membrane and cytoskeletal actin to promote cell mobility, adherence, signal transduction, and activation of tyrosine kinases [47]. As increased metastatic potential and decreased survival have been observed in rhabdomyosarcoma, Ewing's sarcoma, myxofibrosarcoma, chondrosarcoma, etc., it was proposed that targeting ezrin is expected to prevent metastatic progression [2]. Direct inhibitors of ezrin, small molecules NSC305787 and NSC668394, demonstrated statistically significant reduction in tumor growth *in vitro* using the model of osteosarcoma [48]. Moreover, ezrin is a downstream target of SMARCB1/INI1, regulator of chromatin remodeling, and potential tumor suppressor [49]. Inactivating mutation in SMARCB1/INI1 is an oncogene driver in rhabdoid tumors, and the loss of functional SMARCB1/INI1 leads to increase in ezrin expression [50]. *In vitro* and *in vivo* studies demonstrated the efficacy of ezrin inhibitor DZNep in treatment of this subset of STS [50].

Besides the abovementioned pathways and molecules, Notch, Wnt/*β*-catenin, PI3K/Akt/mTOR, VEGF, and other signaling pathways may also promote tumor cell proliferation, survival, migration, angiogenesis, and metastasis in sarcomas. Moreover, multiple studies have already demonstrated the perspectives of the suppression of key components of these cascades for the therapy of STS (Tables 1 and 2). However, reaching therapeutic efficacy of newly developed as well as approved drugs on patient tumors is still challenging and demands additional approaches to find out the effective treatment. Use of chemotherapy sensitivity and resistance assays together with genetic testing could be a step to significant improvement of STS therapy.

### 2.3. Immunotherapy of Soft Tissue Sarcomas

Nowadays, inhibitors of immune system checkpoints are considered as the most promising drug category for many malignances, and they have been already applied in STS therapy also [51]. The expression of ligand of programmed death-1 (PD-L1) and PD-L2 was considered as one of the most important biomarkers for PD-1 inhibitor assignment; a high expression of PD-L1 could be a predictive factor of response to anti-PD-1 therapy, and in some malignancies, such as non-small-cell-lung cancers, the evaluation of PD-L1 expression was inseparably linked to the indication of immunotherapy [52]. The expression of PD-L1 in soft tissue sarcomas has been evaluated, and anti-PD-1 therapies showed clinical evidence of benefit [53]. However, in prospective clinical trials, anti-PD-1 therapy for STS resulted in minimal patient responses [54, 55]. Activation of indoleamine 2,3-dioxygenase 1 (IDO1) by anti-PD-1 therapy could be a new target of combined immunotherapy [56]. Immunotherapy with chimeric antigen receptor-modified T cells (CART) or dendritic cells has been also investigated [52]. Targeted immunotherapy with the cancer-testis antigen NY-ESO-1 for synovial sarcoma has shown especially promising results for patients with a specific human leukocyte antigen (HLA) haplotype, HLA-A∗0201 [57].

A critical challenge of evaluation of immunotherapy application in STS management is the rarity of the disease and heterogeneity of its subtypes. Multicenter preclinical and clinical study collaborations are needed to efficiently enroll enough patients to assess efficacy of specific therapy. It appears that STS subtypes exhibit varying sensitivity to a particular immunotherapeutic strategy. Therefore, clinical trials should ideally be conducted for a specific STS subtype rather than collectively for all STS. Similarly, preclinical research should focus on an understanding of the native immune response and inhibitory mechanisms present in the tumor microenvironment that are unique and specific to each STS subtype. With the differences in STS biology, clinical behavior, and response to therapy, it is very likely that the immune response is also distinct between subtypes of disease. These immunologic differences need to be recognized and appropriately incorporated into the design of immunotherapeutic strategies for each STS subtype.

## 3. Drug Sensitivity Testing on Patient-Derived STS Cells

The majority of the CSRA has been developed during the past 20–30 years. Some of them have been revised, improved, and currently in use in clinical trials. However, none of these assays is in the routine clinical use due to their complex design and still lacking a strong correlation between results of testing *in vitro* and therapeutic outcome *in vivo*. Personalized treatment approaches take into account individual tumor characteristics: oncogenic mutation, chromosomal translocation, specific gene amplifications, and protein expression levels. Personalized CSRA testing could be a further step in identifying the appropriate chemotherapeutics and molecular targeting agents.

Studies describing CSRA in soft tissue sarcoma patients are largely missing. There are multiple studies describing cytotoxicity assays in sarcoma cell lines and anticancer activity *in vivo*, mainly in xenograft models [58–61]. More than 600 established sarcoma cell lines are available for screening, and, as STS is a highly heterogeneous group of cancer, there needs to be even a larger number of cell lines, with various histological subtypes, to better benefit sarcoma research [62].

There are a number of studies based on patient-derived cells (PDC) and patient-derived xenograft (PDX) model *in vivo*. However, most of the described investigations are focused on establishment of new cell line derived from patient tumor (for example, see [63–67], on preclinical studies of novel or repurposed drug/combination of drugs *in vitro* [68–71] and *in vivo* [72–74]). Only a few studies address the optimization of STS treatment. Thus, the efficacy of temozolomide treatment was demonstrated in the PDX model of doxorubicin-resistant undifferentiated spindle-cell sarcoma [75]. Moreover, Igarashi et al. concluded that the PDX model used in the study could identify promising therapies with significantly greater efficacy than first-line therapy for this recalcitrant disease. In another study of predictive models for response to therapy, 29 samples of patient tumors were engrafted in immunodeficient mice (“TumorGraft” method) and 22 (76%) of them were subsequently engrafted in mice for drug sensitivity testing. The most relevant finding was that TumorGraft could predict response to treatment in 13 of 16 cases of sarcoma patients undergoing treatment. The main disadvantage of this model system was the fact that period duration from tumor engraftment to drug sensitivity assay fulfillment was several months [76], which presents a dramatic limitation for patients with progressive disease. However, these results demonstrate that patient-derived sarcoma cells or xenografts are relevant models that can be used to identify effective treatments for sarcoma patients. In line with this study, Brodin et al. performed the genomic profile and drug sensitivity testing of samples from sarcoma patients and showed that drug sensitivity of the patient sarcoma cells *ex vivo* correlated with the response to the actual treatment. ATP-TCA assay was used for evaluation of the viable cell number [77].

A growing evidence suggests that more complex three-dimensional (3D) models are necessary to properly mimic many of the critical hallmarks of soft tissue sarcoma. A number of innovative methods are being studied to fabricate biomimetic sarcoma tumors, encompassing both the surrounding cellular milieu and extracellular matrix. For example, certain advantages were described for 3D models of Ewing's sarcoma [78].

These pilot studies show that patient-derived sarcoma cells can be isolated from biopsies and expanded *in vitro* for drug sensitivity testing. This rapid approach does not require budget- and time-consuming immunodeficient animals and can predict the patient response to standard or experimental treatments. However, trials with larger cohorts need to be performed to confirm its clinical value.

## 4. Conclusion

Given the genetic and histological diversity of this large family of cancers, the treatment of STS calls for a multidisciplinary approach to achieve optimal outcomes. Future studies in the field should be focused on identification of known specific molecular markers in patient tumor tissue, identification and validation of new molecular targets, and validation and prospective use of drug-sensitivity test systems *in vitro*.

## Figures and Tables

**Table 1:** Chromosomal translocations in STS.

Tumor type	Translocation	Fusion product	Targeted therapy approach based on genetic testing	Stage of investigations	Reference
Alveolar rhabdomyosarcoma	t(2; 13) (q35; q14)	PAX3-FOXO1A (aberrant transcription)	Inhibition of regulatory networks (phosphorylation, transcription, coactivation, acetylation)	*In vitro*/*in vivo* studies	[32, 79]
			Inhibition of downstream targets (FGFR4, ALK1, PDGFR-alpha, IGF1R, etc.)	Multiple clinical trials involving FDA-approved drugs (ponatinib, crizotinib, sorafenib, sunitinib, sphingosine, etc.)	[32, 79]
	t(1; 13) (p36; q14)	PAX7-FOXO1A (aberrant transcription)	Not described	Not described	[80]
	t(X; 2) (q13; q35)	PAX3-FOXO4 (aberrant transcription)	Not described	Not described	[81]
	t(2; 2) (q35; p23)	PAX3-NCOA1 (aberrant transcription)	Not described	Not described	[82]
	t(2; 8) (q35; q13.3)	PAX3-NCOA2 (aberrant transcription)	Not described	Not described	[83]
	t(8; 13) (p11; q11)	FOXO1-FGR1 (aberrant transcription)	Not described	Not described	[81]

Alveolar soft part sarcoma	t(X; 17) (p11.2; q25)	TFE3-ASPL (aberrant transcription)	Not described	Not described	[84]

Angiomatoid fibrous histiosarcoma	t(12; 16) (q13; p11)	FUS-ATF1 (aberrant transcription)	Not described	Not described	[85, 86]

Chondroid lipoma	t(11; 16) (q13; p12-13)	C11orf95-MKL2	Not described	Not described	[87]

Clear cell sarcoma	t(12; 22) (q13; q12)	EWS-ATF1 (aberrant transcription)	Inhibition of EWS-ATF1 downstream target c-Met, an oncogenic receptor tyrosine kinase, with small-molecule inhibitor SU11274 or a neutralizing antibody to its ligand HGF AMG 102	*In vitro*/*in vivo* studies	[88]
			Inhibition of EWS-ATF1 downstream target proto-oncogene FOS, with FOS-targeted siRNA	*In vitro*/*in vivo* studies	[89]

Congenital fibrosarcoma/mesoblastic nephroma	t(12; 15) (p13; q25)	ETV6-NTRK3 (ligand-independent kinase activation)	Inhibition of ETV6-NTRK3 with LOXO-101, an experimental, highly selective inhibitor of TRK	Phase I clinical trial	[4]

Dermatofibrosarcoma	t(17; 22) (q22; q13)	COL1A1-PDGFB (increased expression of kinase)	Inhibition of PDGFRB with imatinib	Approval for systemic treatment of dermatofibrosarcoma	[5]
			Inhibition of PDGFRB with sunitinib	Trials for use in case of imatinib-resistant dermatofibrosarcoma	[6, 7]

Desmoplastic small round cell tumor	t(11; 22) (p13; q12)	EWS-WT1 (aberrant transcription)	Inhibition of EWS-WT1 expression with trabectedin	*In vitro*/*in vivo* studies	[38]
			Inhibition of EWS-WT1 downstream target IGF1R with monoclonal antibody ganitumab	Phase II clinical trial completed	[42]
			Inhibition of EWS-WT1 downstream targets (mTOR, Notch, PDGFRB) with known approved inhibitors	Phases I-II are ongoing or completed without significantly improvement of therapy outcomes	[90]

Endometrial stromal sarcoma, low grade	t(7; 17) (p15; q21)	JAZF1-JJAZ1 (aberrant transcription)	Not described	Not described	[91]

Epithelioid hemangioendothelioma	t(1; 3) (p36; q25)	WWTR1-CAMTA1 (aberrant transcription)	Not described	Not described	[81]
	t(X; 11) (p11.2; q13)	YAP1-TFE3 (aberrant transcription)	Not described	Not described	[92]

Ewing sarcoma and peripheral primitive neuroectodermal tumor	t(21; 22) (q22; q12)	EWS-FLI1 (aberrant transcription)	Inhibition of EWS-FLI1 activity with low molecular weight compound YK-4-279	*In vitro*/*in vivo* studies	[19, 93, 94]
			Inhibition of EWS-FLI1 activity with analogues of mythramycin	*In vitro*/*in vivo* studies	[95]
			Inhibition of EWS-FLI1 phosphorylation with EnglerinA	*In vitro* studies	[29]
			Inhibition of EWS-FLI1 expression with antisense oligodeoxynucleotides, siRNAs	*In vitro*/*in vivo* studies	[27, 96, 97]
			Inhibition of EWS-FLI1 downstream target IGF1R with monoclonal antibodies R1507, MK-0646, cixutumumab, Ganitumab, figitumumab	Phase I-II clinical trials	[27]
			Inhibition of EWS-FLI1 downstream targets Aurora kinase (AURK) family members (A, B, and C) with AURKA inhibitors alisertib and tozasertib	*In vitro*/*in vivo* studies Phase I clinical trial did not demonstrated high efficacy	[27]
			Inhibition of EWS-FLI1 activity and its downstream targets with trabectedin and its analogues	*In vitro*/*in vivo* studies Phase I-II clinical trials of Trabectedin-based combined chemotherapy	[40, 98–101]
			Inhibition of EWS-FLI1 downstream target CDK7/12/13 with low molecular weight CDK7/12/13 inhibitor THZ1/THZ531	*In vitro*/*in vivo* studies	[102]
			Inhibition of EWS-FLI1 using PARP inhibitor olaparib	*In vitro*/*in vivo* studies	[30, 31]
			Inhibition of EWS-FLI1 using HDAC and DMT inhibitors	*In vitro*/*in vivo* studies	[27]
			Inhibition of EWS-FLI1 using combination therapy with PARP inhibitors and trabectedin	*In vitro/in vivo* studies	[103, 104]
	t(11; 22) (q24; q12)	EWS-ERG (aberrant transcription)	Same approaches as in case of EWS-FLI1-positive disease could be used as differences in the C-terminal partner in gene fusions are not associated with significant phenotypic differences	*In vitro*/*in vivo* studies	[105, 106]
	t(17; 22) (q12; q12)	EWS-E1AF (aberrant transcription)	Not described	Not described	[107]
	t(2; 22) (q33; q12)	EWS-FEV (aberrant transcription)	Not described	Not described	[108]
	t(7; 22) (p22; q12)	EWS-ETV1 (aberrant transcription)	Not described	Not described	[109]
	t(17; 22) (q21; q12)	EWS-ETV4 (aberrant transcription)	Not described	Not described	[81]
	inv(22) (q12; q12)	EWS-PATZ1 (aberrant transcription)	Not described	Not described	[81]
	t(2; 22) (q31; q12)	EWS-SP3 (aberrant transcription)	Not described	Not described	[110]
	t(20; 22) (q13; q12)	EWS-NFATC2 (aberrant transcription)	Not described	Not described	[81]
	t(4; 22) (q31; q12)	EWS-SMARCA5 (aberrant transcription)	Not described	Not described	[111]
	t(16; 21) (p11; q22)	FUS-ERG (aberrant transcription)	Inhibition of FUS-ERG downstream targets CDK4/6 and IGFR1 with linsitinib and palbociclib	*In vitro*/*in vivo* studies	[112]
	t(2; 16) (q36; p11)	FUS-FEV (aberrant transcription)	Not described	Not described	[81]

Extraskeletal myxoid chondrosarcoma	t(9; 17) (q22; q11)	RBPP56-NR4A3 (aberrant transcription)	Not described	Not described	[113]
	t(9; 15) (q22; q21)	TCF12-NR4A3 (aberrant transcription)	Not described	Not described	[114]
	t(2; 22) (q34; q12)	EWS-FEV (aberrant transcription)	Not described	Not described	30776935
	t(9; 22) (q22; q12)	EWS-NR4A3 (aberrant transcription)	Correlation in survival after sunitinib-based therapy and the presence of EWS-NR4A3 translocation	Clinical report	[115, 116]

Fibromyxoid sarcoma, low grade	t(7; 16) (q33; p11)	FUS-CREB3L2 (aberrant transcription)	Inhibition of FUS-CREB3L2 and FUS-CREB3L1 potential downstream target CD24	*In silico* studies	[117]
	t(11; 16) (p11; p11)	FUS-CREB3L1 (rare) (aberrant transcription)			
	t(11; 22) (q11; q12)	EWS- CREB3L1 (aberrant transcription)	Not described	Not described	[118]

Glomus tumor	t(1; 5) (p13; q32)	MIR143-NOTCH2 (aberrant transcription)	Not described	Not described	[81]
	t(5; 9) (q32; q34.3)	MIR143-NOTCH1 (aberrant transcription)	Not described	Not described	[81]

Inflammatory myofibroblastic tumor	t(2; 19) (p23; p13.1)	TPM4-ALK (aberrant transcription)	Inhibition of the expression of ALK fusion genes by low molecular weight compounds of natural and synthetic origin Inhibition of the expression of ALK fusion genes by ALK inhibitor crizotinib	*In silico*/*in vitro* studies Phase II-III clinical trials	[23] [22]
	t(1; 2) (q22-23; p23)	TPM3-ALK (aberrant transcription)			
	t(2; 17) (p23; q23)	CLTC-ALK (aberrant transcription)			
	t(2; 2) (p23; q23)	RANBP2-ALK (aberrant transcription)			
	t(2; 11) (p23; p15)	CARS-ALK (aberrant transcription)			
	inv(2) (p23; q35)	ATIC-ALK (aberrant transcription)			
	t(2; 4) (p23; q21)	SEC31A-ALK (aberrant transcription)			
	t(2; 12) (p23; p12)	PPFIBP1-ALK (aberrant transcription)			

Mesenchymal chondrosarcoma	t(8; 8) (q13; q21)	HEY1-NCOA2 (aberrant transcription)	Not described	Not described	[119]


Myoepithelial tumors	t(6; 22) (p21; q12)	EWS-POU5F1 (aberrant transcription)	Not described	Not described	[120]
	t(19; 22) (q13; q12)	EWS-ZNF444 (aberrant transcription)	Not described	Not described	[81]
	t(1; 22) (q23; q12)	EWS-PBX1 (aberrant transcription)	Not described	Not described	[81]

Myxoinflammatory fibroblastic sarcoma/hemosiderotic fibrolipomatous tumor	t(1; 10) (p33; q34) and amplification of 3p11-12	TGFBR3-MGFA5 (amplification of VGLL3)	Not described	Not described	[81]

Myxoid liposarcoma	t(12; 16) (q13; p13)	FUS-DDIT3 (aberrant transcription)	Inhibition of FUS-DDIT3 expression with siRNAs	*In vitro* studies	[121]
			Inhibition of FUS-DDIT3 activity by direct binding of Trabectedin	*In vitro* studies	[39]
			Inhibition of FUS-DDIT3 downstream targets IGF-IR/PI3K/Akt with their known inhibitors	*In vitro* studies	[121]
	t(12; 22) (q13; q11-q12)	EWS-DDIT3 (aberrant transcription)	Inhibition of EWS-DDIT3 activity by direct binding of Trabectedin	*In vitro* studies	[39]

Nodular fascitis	t(17; 22) (p13; q13)	MYH9-USP6 (aberrant transcription)	Not described	Not described	[122]

Ossifying fibromyxoid tumor	t(6; 12) (p21.2; q24.33)	EP400-PHF1 (aberrant transcription)	Not described	Not described	[123]
	t(1; 6) (p34.3; p21.2)	MEAF6-PHF1 (aberrant transcription)	Not described	Not described	[124]
	t(X; 22) (p11; q13)	ZC3H7B-BCOR (aberrant transcription)	Not described	Not described	[123]
	t(6; 10) (p21.2; p11)	EPC1-PHF1 (aberrant transcription)	Not described	Not described	[123]

Pericytoma	t(7; 12) (p22; q13)	ACTB-GLI1 (aberrant transcription)	Not described	Not described	[125]

Pseudomyogenic hemangioendothelioma	t(7; 19) (q22; q13)	SERPINE1-FOSB (aberrant transcription)	Inhibition of SERPINE1 with VEGFR1-4/PDGFRA inhibitor telatinib	*In vitro* studies	[126]

Sclerosing epithelioid fibrosarcoma	t(7; 16) (q34; p11)	FUS-CREB3L2 (aberrant transcription)	Inhibition of FUS-CREB3L2 potential downstream target CD24	*In silico* studies	[117]

Soft tissue angiofibroma	t(5; 8) (p15; q13)	AHRR-NCOA2 (aberrant transcription)	Not described	Not described	[127]

Solitary fibrous tumor	12q13(inversion)	NAB2-STAT6 (aberrant transcription)	Not described	Not described	[128]

Congenital/infantile spindle cell rhabdomyosarcoma	t(2; 8) (q35; q13)	PAX3-NCOA2 (aberrant transcription)	Not described	Not described	[83]
	t(6; 8) (p12; q13)	SRF-NCOA2 (aberrant transcription)	Not described	Not described	[129]
	t(8; 11) (q13; p15)	TEAD1-NCOA2 (aberrant transcription)	Not described	Not described	[130]

Synovial sarcoma	t(X; 18) (p11; q11)	SS18-SSX1 (aberrant transcription)	Inhibition of SS18-SSX1expression with siRNAs	*In vitro*/*in vivo* studies	[28, 131]
			Inhibition of SS18-SSX1 downstream signaling pathways VEGFA, IGFR1, Wnt/b-catenin and chromatine remodeling proteins with their known inhibitors (Wnt inhibitor monoclonal antibody FZD10, IGFR1 inhibitor cixutumumab, VEGFA inhibitor bevacizumab, HDAC inhibitors, trabectedin and sorafenib for multiple pathways)	Phase I-II clinical trials	[132]
		SS18-SSX2 (aberrant transcription)	Inhibition of SS18-SSX2 downstream signaling pathways VEGFA, IGFR1, Wnt/b-catenin, and chromatin remodeling proteins with their known inhibitors (Wnt inhibitor monoclonal antibody FZD10, IGFR1 inhibitor cixutumumab, VEGFA inhibitor bevacizumab, HDAC inhibitors, trabectedin and sorafenib for multiple pathways)	Phase I-II clinical trials	[132]
		SS18-SSX4 (rare) (aberrant transcription)	Inhibition of SS18-SSX2 downstream signaling pathways VEGFA, IGFR1, Wnt/b-catenin, and chromatin remodeling proteins with their known inhibitors (Wnt inhibitor monoclonal antibody FZD10, IGFR1 inhibitor cixutumumab, VEGFA inhibitor bevacizumab, HDAC inhibitors, trabectedin and sorafenib for multiple pathways)	Phase I-II clinical trials	[132]

Tenosinovial giant cell tumor	t(1; 2) (p13; q35-37)	COL6A3-CSF1 (aberrant transcription)	Not described	Not described	[133]

Undifferentiated round cell sarcoma	t(4; 19) (q35; q13)	CIC-DUX4 (aberrant transcription)	Not described	Not described	[134]
	Xp11 (inversion)	BCOR-CCNB3 (aberrant transcription)	Not described	Not described	[135]

**Table 2:** Oncogenic mutations and gene amplifications in STS.

Tumor type	Gene	Therapy approach	Stage of investigations	Reference
*Activating mutations*				
Gastrointestinal stromal tumor	c-KIT	Inhibition of c-KIT with imatinib, nilotinib, and pazopanib	Phase I-III clinical trials	[52, 136, 137]
	PDGFRA	Inhibition of PDGFRA with olaratumab, imatinib, pazopanib, regorafenib, sorafenib, and sunitinib	Phase I-II clinical trials	[138–141]
	BRAF	Inhibition of BRAF with dabrafenib and vemurafenib	*In vitro*/*in vivo* studies; clinical case report	[142, 143]
Myxoid round cell liposarcoma	PI3CA	Inhibition of PI3K/Akt signaling with multiple known inhibitors	*In vitro*/*in vivo* studies	[144, 145]
*Inactivating mutations*				
Malignant peripheral nerve sheath tumor	NF-1	Inhibition of NF-1 downstream Ras-dependent targets Src kinase (CGP77675) and MEK-1 (U0126)	*In vitro*/*in vivo* studies	[146]
		Inhibition of NF-1 downstream target mTOR signaling pathway by temsirolimus, everolimus, and sirolimus	*In vitro*/*in vivo* studies	[147]
Rhabdoid tumors	INI1	Inhibition of INI1 downstream targets, epigenetic regulators HDACs, EZH2, and eIF2α with their known inhibitors	*In vitro*/*in vivo* studies	[50]
Perivascular epithelioid cell tumors	TSC1/2	Inhibition of mTOR signaling with known mTOR inhibitors temsirolimus, ridaforolimus, everolimus, and sirolimus	Phase I-II clinical trials	[24]
*Gene amplifications*				
Dedifferentiated and well-differentiated liposarcoma	MDM2	Inhibition of MDM2 with antagonist RG7388	*In vitro*/*in vivo* studies	[26]
	CDK4	Inhibition of CDK4 with palbociclib	Phase I-II clinical trials	[25]
	c-JUN	Not described	Not described	[148]
Intimal sarcomas	MDM2	Inhibition of MDM2 with antagonist RG7388	*In vitro*/*in vivo* studies	[26]
	CDK4	Inhibition of CDK4 with palbociclib	Phase I-II clinical trials	[25]

## References

[1] Jemal A., Siegel R., Xu J., Ward E. (2010). Cancer statistics, 2010. *CA: A Cancer Journal for Clinicians*.

[2] Harwood J. L., Alexander J. H., Mayerson J. L., Scharschmidt T. J. (2015). Targeted chemotherapy in bone and soft-tissue sarcoma. *Orthopedic Clinics of North America*.

[3] Gronchi A., Maki R. G., Jones R. L. (2017). Treatment of soft tissue sarcoma: a focus on earlier stages. *Future Oncology*.

[4] Nagasubramanian R., Wei J., Gordon P., Rastatter J. C., Cox M. C., Pappo A. (2016). Infantile fibrosarcoma WithNTRK3-ETV6 fusion successfully treated with the tropomyosin-related kinase inhibitor LOXO-101. *Pediatric Blood & Cancer*.

[5] Ugurel S., Kortmann R. D., Mohr P. (2019). S1-leitlinie dermatofibrosarcoma protuberans (DFSP)—update 2018. *JDDG: Journal der Deutschen Dermatologischen Gesellschaft*.

[6] Xiao W., Que Y., Peng R. (2018). A favorable outcome of advanced dermatofibrosarcoma protuberans under treatment with sunitinib after imatinib failure. *OncoTargets and Therapy*.

[7] Fu Y., Kang H., Zhao H. (2015). Sunitinib for patients with locally advanced or distantly metastatic dermatofibrosarcoma protuberans but resistant to imatinib. *International Journal of Clinical and Experimental Medicine*.

[8] Monk B. J., Herzog T. J., Tewari K. S. (2016). Evolution of chemosensitivity and resistance assays as predictors of clinical outcomes in epithelial ovarian cancer patients. *Current Pharmaceutical Design*.

[9] Schrag D., Garewal H. S., Burstein H. J. (2004). American society of clinical oncology technology assessment: chemotherapy sensitivity and resistance assays. *Journal of Clinical Oncology*.

[10] Pauli C., Hopkins B. D., Prandi D. (2017). Personalized in vitro and in vivo cancer models to guide precision medicine. *Cancer Discovery*.

[11] Bruna A., Rueda O. M., Caldas C. (2016). Modeling breast cancer intertumor and intratumor heterogeneity using xenografts. *Cold Spring Harbor Symposia on Quantitative Biology*.

[12] Gunby R., Sala E., Tartari C., Puttini M., Gambacorti-Passerini C., Mologni L. (2007). Oncogenic fusion tyrosine kinases as molecular targets for anti-cancer therapy. *Anti-Cancer Agents in Medicinal Chemistry*.

[13] Redell M. S., Tweardy D. J. (2006). Targeting transcription factors in cancer: challenges and evolving strategies. *Drug Discovery Today: Technologies*.

[14] Dang C. V., Reddy E. P., Shokat K. M., Soucek L. (2017). Drugging the “undruggable” cancer targets. *Nature Reviews Cancer*.

[15] Orbach D., Brennan B., De Paoli A. (2016). Conservative strategy in infantile fibrosarcoma is possible: the European paediatric soft tissue sarcoma study group experience. *European Journal of Cancer*.

[16] Lannon C. L., Sorensen P. H. B. (2005). ETV6-NTRK3: a chimeric protein tyrosine kinase with transformation activity in multiple cell lineages. *Seminars in Cancer Biology*.

[17] Cidre-Aranaz F., Alonso J. (2015). EWS/FLI1 target genes and therapeutic opportunities in ewing sarcoma. *Frontiers in Oncology*.

[18] Fidaleo M., De Paola E., Paronetto M. P. (2016). The RNA helicase A in malignant transformation. *Oncotarget*.

[19] Kovar H. (2014). Blocking the road, stopping the engine or killing the driver? Advances in targeting EWS/FLI-1 fusion in Ewing sarcoma as novel therapy. *Expert Opinion on Therapeutic Targets*.

[20] Hoang N. T., Acevedo L. A., Mann M. J., Tolani B. (2018). A review of soft-tissue sarcomas: translation of biological advances into treatment measures. *Cancer Management and Research*.

[21] Antonescu C. R. (2006). The role of genetic testing in soft tissue sarcoma. *Histopathology*.

[22] Mossé Y. P., Voss S. D., Lim M. S. (2017). Targeting ALK with Crizotinib in pediatric anaplastic large cell lymphoma and inflammatory myofibroblastic tumor: a children’s oncology group study. *Journal of Clinical Oncology*.

[23] Li R., Morris S. W. (2008). Development of anaplastic lymphoma kinase (ALK) small-molecule inhibitors for cancer therapy. *Medicinal Research Reviews*.

[24] Linch M., Miah A. B., Thway K., Judson I. R., Benson C. (2014). Systemic treatment of soft-tissue sarcoma-gold standard and novel therapies. *Nature Reviews Clinical Oncology*.

[25] Martin-Liberal J., Pérez E., García Del Muro X. (2019). Investigational therapies in phase II clinical trials for the treatment of soft tissue sarcoma. *Expert Opinion on Investigational Drugs*.

[26] Laroche-Clary A., Chaire V., Algeo M.-P., Derieppe M.-A., Loarer F. L., Italiano A. (2017). Combined targeting of MDM2 and CDK4 is synergistic in dedifferentiated liposarcomas. *Journal of Hematology & Oncology*.

[27] Yu H., Ge Y., Guo L., Huang L. (2017). Potential approaches to the treatment of Ewing’s sarcoma. *Oncotarget*.

[28] Takenaka S., Naka N., Araki N. (2010). Downregulation of SS18-SSX1 expression in synovial sarcoma by small interfering RNA enhances the focal adhesion pathway and inhibits anchorage-independent growth in vitro and tumor growth in vivo. *International Journal of Oncology*.

[29] Caropreso V., Darvishi E., Turbyville T. J. (2016). Englerin A inhibits EWS-FLI1 DNA binding in ewing sarcoma cells. *Journal of Biological Chemistry*.

[30] Lee H.-J., Yoon C., Schmidt B. (2013). Combining PARP-1 inhibition and radiation in Ewing sarcoma results in lethal DNA damage. *Molecular Cancer Therapeutics*.

[31] Engert F., Schneider C., Weiss L. M., Probst M., Fulda S. (2015). PARP inhibitors sensitize ewing sarcoma cells to temozolomide-induced apoptosis via the mitochondrial pathway. *Molecular Cancer Therapeutics*.

[32] Nguyen T. H., Barr F. G. (2018). Therapeutic approaches targeting PAX3-FOXO1 and its regulatory and transcriptional pathways in rhabdomyosarcoma. *Molecules*.

[33] Pantziarka P., Bouche G., Meheus L., Sukhatme V., Sukhatme V. P., Vikas P. (2014). The repurposing drugs in oncology (ReDO) project. *Ecancermedicalscience*.

[34] Yen C.-C., Chen T. W.-W. (2018). Next frontiers in systemic therapy for soft tissue sarcoma. *Chinese Clinical Oncology*.

[35] Cuevas C., Francesch A. (2009). Development of Yondelis (trabectedin, ET-743): a semisynthetic process solves the supply problem. *Natural Product Reports*.

[36] Larsen A. K., Galmarini C. M., D’Incalci M. (2016). Unique features of trabectedin mechanism of action. *Cancer Chemotherapy and Pharmacology*.

[37] Uboldi S., Calura E., Beltrame L. (2012). A systems biology approach to characterize the regulatory networks leading to trabectedin resistance in an in vitro model of myxoid liposarcoma. *PLoS One*.

[38] Uboldi S., Craparotta I., Colella G. (2017). Mechanism of action of trabectedin in desmoplastic small round cell tumor cells. *BMC Cancer*.

[39] Ganjoo K. N., Patel S. (2009). Trabectedin: an anticancer drug from the sea. *Expert Opinion on Pharmacotherapy*.

[40] Grohar P. J., Segars L. E., Yeung C. (2014). Dual targeting of EWS-FLI1 activity and the associated DNA damage response with trabectedin and SN38 synergistically inhibits ewing sarcoma cell growth. *Clinical Cancer Research*.

[41] Garofalo C., Manara M. C., Nicoletti G. (2011). Efficacy of and resistance to anti-IGF-1R therapies in Ewing’s sarcoma is dependent on insulin receptor signaling. *Oncogene*.

[42] Tap W. D., Demetri G., Barnette P. (2012). Phase II study of Ganitumab, a fully human anti-type-1 insulin-like growth factor receptor antibody, in patients with metastatic ewing family tumors or desmoplastic small round cell tumors. *Journal of Clinical Oncology*.

[43] Mehta C. R., Liu L., Theuer C. (2019). An adaptive population enrichment phase III trial of TRC105 and pazopanib versus pazopanib alone in patients with advanced angiosarcoma (TAPPAS trial). *Annals of Oncology*.

[44] Puerto-Camacho P., Amaral A. T., Lamhamedi-Cherradi S.-E. (2019). Preclinical efficacy of endoglin-targeting antibody-drug conjugates for the treatment of ewing sarcoma. *Clinical Cancer Research*.

[45] Wakahara K., Ohno T., Kimura M. (2008). EWS-Fli1 up-regulates expression of the Aurora A and Aurora B kinases. *Molecular Cancer Research*.

[46] Humme D., Haider A., Möbs M. (2015). Aurora kinase A is upregulated in cutaneous T-cell lymphoma and represents a potential therapeutic target. *Journal of Investigative Dermatology*.

[47] Clucas J., Valderrama F. (2014). ERM proteins in cancer progression. *Journal of Cell Science*.

[48] Bulut G., Hong S.-H., Chen K. (2012). Small molecule inhibitors of ezrin inhibit the invasive phenotype of osteosarcoma cells. *Oncogene*.

[49] Kohashi K., Oda Y. (2017). Oncogenic roles of SMARCB1/INI1 and its deficient tumors. *Cancer Science*.

[50] Moreno N., Kerl K. (2016). Preclinical evaluation of combined targeted approaches in malignant rhabdoid tumors. *Anticancer Research*.

[51] Postow M. A., Callahan M. K., Wolchok J. D. (2015). Immune checkpoint blockade in cancer therapy. *Journal of Clinical Oncology*.

[52] Nakano K., Takahashi S. (2018). Current molecular targeted therapies for bone and soft tissue sarcomas. *International Journal of Molecular Sciences*.

[53] Kim C., Kim E. K., Jung H. (2016). Prognostic implications of PD-L1 expression in patients with soft tissue sarcoma. *BMC Cancer*.

[54] Tawbi H. A., Burgess M., Bolejack V. (2017). Pembrolizumab in advanced soft-tissue sarcoma and bone sarcoma (SARC028): a multicentre, two-cohort, single-arm, open-label, phase 2 trial. *The Lancet Oncology*.

[55] Paoluzzi L., Cacavio A., Ghesani M. (2016). Response to anti-PD1 therapy with nivolumab in metastatic sarcomas. *Clinical Sarcoma Research*.

[56] Toulmonde M., Penel N., Adam J. (2018). Use of PD-1 targeting, macrophage infiltration, and Ido pathway activation in sarcomas. *JAMA Oncology*.

[57] Robbins P. F., Kassim S. H., Tran T. L. N. (2015). A pilot trial using lymphocytes genetically engineered with an NY-ESO-1-reactive T-cell receptor: long-term follow-up and correlates with response. *Clinical Cancer Research*.

[58] Wu M.-H., Lee C.-Y., Huang T.-J. (2018). MLN4924, a protein neddylation inhibitor, suppresses the growth of human chondrosarcoma through inhibiting cell proliferation and inducing endoplasmic reticulum stress-related apoptosis. *International Journal of Molecular Sciences*.

[59] Yahuafai J., Asai T., Oku N., Siripong P. (2018). Anticancer efficacy of the combination of berberine and PEGylated liposomal doxorubicin in meth A sarcoma-bearing mice. *Biological and Pharmaceutical Bulletin*.

[60] Lacroix J., Kis Z., Josupeit R. (2018). Preclinical testing of an oncolytic parvovirus in Ewing sarcoma: protoparvovirus H-1 induces apoptosis and lytic infection in vitro but fails to improve survival in vivo. *Viruses*.

[61] Çelik H., Sciandra M., Flashner B. (2018). Clofarabine inhibits Ewing sarcoma growth through a novel molecular mechanism involving direct binding to CD99. *Oncogene*.

[62] Hattori E., Oyama R., Kondo T. (2019). Systematic review of the current status of human sarcoma cell lines. *Cells*.

[63] Oyama R., Kito F., Qiao Z. (2019). Establishment of novel patient-derived models of dermatofibrosarcoma protuberans: two cell lines, NCC-DFSP1-C1 and NCC-DFSP2-C1. *In Vitro Cellular & Developmental Biology—Animal*.

[64] Oyama R., Kito F., Qiao Z. (2018). Establishment of a novel patient-derived Ewing’s sarcoma cell line, NCC-ES1-C1. *In Vitro Cellular & Developmental Biology—Animal*.

[65] Oyama R., Takahashi M., Kito F. (2018). Establishment and characterization of patient-derived xenograft and its cell line of primary leiomyosarcoma of bone. *In Vitro Cellular & Developmental Biology—Animal*.

[66] De Vita A., Mercatali L., Miserocchi G. (2018). Establishment of a primary culture of patient-derived soft tissue sarcoma. *Journal of Visualized Experiments*.

[67] Kito F., Oyama R., Takai Y. (2018). Establishment and characterization of the NCC-SS1-C1 synovial sarcoma cell line. *Human Cell*.

[68] Kim H. K., Kim S. Y., Lee S. J. (2016). BEZ235 (PIK3/mTOR inhibitor) overcomes pazopanib resistance in patient-derived refractory soft tissue sarcoma cells. *Translational Oncology*.

[69] Bukchin A., Pascual-Pasto G., Cuadrado-Vilanova M. (2018). Glucosylated nanomicelles target glucose-avid pediatric patient-derived sarcomas. *Journal of Controlled Release*.

[70] Di Martile M., Desideri M., Tupone M. G. (2018). Histone deacetylase inhibitor ITF2357 leads to apoptosis and enhances doxorubicin cytotoxicity in preclinical models of human sarcoma. *Oncogenesis*.

[71] Igarashi K., Kawaguchi K., Kiyuna T. (2017). Efficacy in vitro of caffeine and valproic acid on patient-derived undifferentiated pleomorphic sarcoma and rhabdomyosarcoma cell lines. *Anticancer Research*.

[72] Stacchiotti S., Zuco V., Tortoreto M. (2019). Comparative assessment of antitumor effects and autophagy induction as a resistance mechanism by cytotoxics and EZH2 inhibition in INI1-negative epithelioid sarcoma patient-derived xenograft. *Cancers (Basel)*.

[73] Hoffman R. M., Murakami T., Kawaguchi K. (2019). High efficacy of recombinant methioninase on patient-derived orthotopic xenograft (PDOX) mouse models of cancer. *Methods in Molecular Biology*.

[74] Oshiro H., Kiyuna T., Tome Y. (2019). Detection of metastasis in a patient-derived orthotopic xenograft (PDOX) model of undifferentiated pleomorphic sarcoma with red fluorescent protein. *Anticancer Research*.

[75] Igarashi K., Kawaguchi K., Kiyuna T. (2018). Temozolomide regresses a doxorubicin-resistant undifferentiated spindle-cell sarcoma patient-derived orthotopic xenograft (PDOX): precision-oncology nude-mouse model matching the patient with effective therapy. *Journal of Cellular Biochemistry*.

[76] Stebbing J., Paz K., Schwartz G. K. (2014). Patient-derived xenografts for individualized care in advanced sarcoma. *Cancer*.

[77] Brodin B. A., Wennerberg K., Lidbrink E. (2019). Drug sensitivity testing on patient-derived sarcoma cells predicts patient response to treatment and identifies c-Sarc inhibitors as active drugs for translocation sarcomas. *British Journal of Cancer*.

[78] Lamhamedi-Cherradi S.-E., Santoro M., Ramammoorthy V. (2014). 3D tissue-engineered model of Ewing’s sarcoma. *Advanced Drug Delivery Reviews*.

[79] Olanich M. E., Barr F. G. (2013). A call to ARMS: targeting the PAX3-FOXO1 gene in alveolar rhabdomyosarcoma. *Expert Opinion on Therapeutic Targets*.

[80] Fitzgerald J. C., Scherr A. M., Barr F. G. (2000). Structural analysis of PAX7 rearrangements in alveolar rhabdomyosarcoma. *Cancer Genetics and Cytogenetics*.

[81] Hameed M. (2014). Molecular diagnosis of soft tissue neoplasia: clinical applications and recent advances. *Expert Review of Molecular Diagnostics*.

[82] Sumegi J., Streblow R., Frayer R. W. (2009). Recurrent t(2;2) and t(2;8) translocations in rhabdomyosarcoma without the canonical PAX-FOXO1 fuse PAX3 to members of the nuclear receptor transcriptional coactivator family. *Genes Chromosomes Cancer*.

[83] Yoshida H., Miyachi M., Sakamoto K. (2014). PAX3-NCOA2 fusion gene has a dual role in promoting the proliferation and inhibiting the myogenic differentiation of rhabdomyosarcoma cells. *Oncogene*.

[84] Ladanyi M., Lui M. Y., Antonescu C. R. (2001). The der(17)t(X;17)(p11;q25) of human alveolar soft part sarcoma fuses the TFE3 transcription factor gene to ASPL, a novel gene at 17q25. *Oncogene*.

[85] Waters B. L., Panagopoulos I., Allen E. F. (2000). Genetic characterization of angiomatoid fibrous histiocytoma identifies fusion of the FUS and ATF-1 genes induced by a chromosomal translocation involving bands 12q13 and 16p11. *Cancer Genetics and Cytogenetics*.

[86] Raddaoui E., Donner L. R., Panagopoulos I. (2002). Fusion of the FUS and ATF1 genes in a large, deep-seated angiomatoid fibrous histiocytoma. *Diagnostic Molecular Pathology*.

[87] Flucke U., Tops B. B. J., de Saint Aubain Somerhausen N. (2013). Presence of C11 or f95-MKL2 fusion is a consistent finding in chondroid lipomas: a study of eight cases. *Histopathology*.

[88] Davis I. J., McFadden A. W., Zhang Y. (2010). Identification of the receptor tyrosine kinase c-Met and its ligand, hepatocyte growth factor, as therapeutic targets in clear cell sarcoma. *Cancer Research*.

[89] Yamada K., Ohno T., Aoki H. (2013). EWS/ATF1 expression induces sarcomas from neural crest-derived cells in mice. *Journal of Clinical Investigation*.

[90] Bulbul A., Fahy B. N., Xiu J. (2017). Desmoplastic small round blue cell tumor: a review of treatment and potential therapeutic genomic alterations. *Sarcoma*.

[91] Koontz J. I., Soreng A. L., Nucci M. (2001). Frequent fusion of the JAZF1 and JJAZ1 genes in endometrial stromal tumors. *Proceedings of the National Academy of Sciences*.

[92] Kuo F.-Y., Huang H.-Y., Chen C.-L., Eng H.-L., Huang C.-C. (2017). TFE3- rearranged hepatic epithelioid hemangioendothelioma—a case report with immunohistochemical and molecular study. *APMIS*.

[93] Todd R., Lunec J. (2008). Molecular pathology and potential therapeutic targets in soft-tissue sarcoma. *Expert Review of Anticancer Therapy*.

[94] Barber-Rotenberg J. S., Selvanathan S. P., Kong Y. (2012). Single enantiomer of YK-4-279 demonstrates specificity in targeting the oncogene EWS-FLI1. *Oncotarget*.

[95] Osgood C. L., Maloney N., Kidd C. G. (2016). Identification of mithramycin analogues with improved targeting of the EWS-FLI1 transcription factor. *Clinical Cancer Research*.

[96] Rao D. D., Jay C., Wang Z. (2016). Preclinical justification of pbi-shRNA EWS/FLI1 lipoplex (LPX) treatment for Ewing’s sarcoma. *Molecular Therapy*.

[97] Maksimenko A., Malvy C. (2005). Oncogene-targeted antisense oligonucleotides for the treatment of Ewing sarcoma. *Expert Opinion on Therapeutic Targets*.

[98] Amaral A. T., Garofalo C., Frapolli R. (2015). Trabectedin efficacy in Ewing sarcoma is greatly increased by combination with anti-IGF signaling agents. *Clinical Cancer Research*.

[99] Tancredi R., Zambelli A., DaPrada G. A. (2015). Targeting the EWS-FLI1 transcription factor in Ewing sarcoma. *Cancer Chemotherapy and Pharmacology*.

[100] Harlow M. L., Maloney N., Roland J. (2016). Lurbinectedin inactivates the Ewing sarcoma oncoprotein EWS-FLI1 by redistributing it within the nucleus. *Cancer Research*.

[101] Herzog J., von Klot-Heydenfeldt F., Jabar S. (2016). Trabectedin followed by irinotecan can stabilize disease in advanced translocation-positive sarcomas with acceptable toxicity. *Sarcoma*.

[102] Iniguez A. B., Stolte B., Wang E. J. (2018). EWS/FLI confers tumor cell synthetic lethality to CDK12 inhibition in Ewing sarcoma. *Cancer Cell*.

[103] Stewart E., Goshorn R., Bradley C. (2014). Targeting the DNA repair pathway in Ewing sarcoma. *Cell Reports*.

[104] Ordóñez J. L., Amaral A. T., Carcaboso A. M. (2015). The PARP inhibitor olaparib enhances the sensitivity of Ewing sarcoma to trabectedin. *Oncotarget*.

[105] Ginsberg J. P., de Alava E., Ladanyi M. (1999). EWS-FLI1 and EWS-ERG gene fusions are associated with similar clinical phenotypes in Ewing’s sarcoma. *Journal of Clinical Oncology*.

[106] Asami S., Chin M., Shichino H. (2008). Treatment of Ewing’s sarcoma using an antisense oligodeoxynucleotide to regulate the cell cycle. *Biological & Pharmaceutical Bulletin*.

[107] Kaneko Y., Yoshida K., Handa M. (1996). Fusion of anETS-family gene, EIAF, to EWS by t(17;22)(q12;q12) chromosome translocation in an undifferentiated sarcoma of infancy. *Genes, Chromosomes and Cancer*.

[108] Llombart-Bosch A., Pellín A., Carda C., Noguera R., Navarro S., Peydró-Olaya A. (2000). Soft tissue Ewing sarcoma-peripheral primitive neuroectodermal tumor with atypical clear cell pattern shows a new type of EWS-FEV fusion transcript. *Diagnostic Molecular Pathology*.

[109] Shulman S. C., Katzenstein H., Bridge J. (2012). Ewing sarcoma with 7;22 translocation: three new cases and clinicopathological characterization. *Fetal and Pediatric Pathology*.

[110] Wang L., Bhargava R., Zheng T. (2007). Undifferentiated small round cell sarcomas with rare EWS gene fusions. *The Journal of Molecular Diagnostics*.

[111] Sumegi J., Nishio J., Nelson M., Frayer R. W., Perry D., Bridge J. A. (2011). A novel t(4;22)(q31;q12) produces an EWSR1-SMARCA5 fusion in extraskeletal Ewing sarcoma/primitive neuroectodermal tumor. *Modern Pathology*.

[112] Murakami T., Singh A. S., Kiyuna T. (2016). Effective molecular targeting of CDK4/6 and IGF-1R in a rare FUS-ERG fusion CDKN2A-deletion doxorubicin-resistant Ewing’s sarcoma patient-derived orthotopic xenograft (PDOX) nude-mouse model. *Oncotarget*.

[113] Sjögren H., Meis-Kindblom J., Kindblom L. G., Aman P., Stenman G. (1999). Fusion of the EWS-related gene TAF2N to TEC in extraskeletal myxoid chondrosarcoma. *Cancer Research*.

[114] Sjögren H., Wedell B., Meis-Kindblom J. M., Kindblom L. G., Stenman G., Kindblom J. M. (2000). Fusion of the NH2-terminal domain of the basic helix-loop-helix protein TCF12 to TEC in extraskeletal myxoid chondrosarcoma with translocation t(9;15)(q22;q21). *Cancer Research*.

[115] Labelle Y., Zucman J., Stenman G. (1995). Oncogenic conversion of a novel orphan nuclear receptor by chromosome translocation. *Human Molecular Genetics*.

[116] Stacchiotti S., Pantaleo M. A., Astolfi A. (2014). Activity of sunitinib in extraskeletal myxoid chondrosarcoma. *European Journal of Cancer*.

[117] Arbajian E., Puls F., Antonescu C. R. (2017). In-depth genetic analysis of sclerosing epithelioid fibrosarcoma reveals recurrent genomic alterations and potential treatment targets. *Clinical Cancer Research*.

[118] Ertoy Baydar D., Kosemehmetoglu K., Aydin O., Bridge J. A., Buyukeren B., Aki F. T. (2015). Primary sclerosing epithelioid fibrosarcoma of kidney with variant histomorphologic features: report of 2 cases and review of the literature. *Diagnostic Pathology*.

[119] Cohen J. N., Solomon D. A., Horvai A. E., Kakar S. (2016). Pancreatic involvement by mesenchymal chondrosarcoma harboring the HEY1-NCOA2 gene fusion. *Human Pathology*.

[120] Fujino T., Nomura K., Ishikawa Y. (2010). Function of EWS-POU5F1 in sarcomagenesis and tumor cell maintenance. *The American Journal of Pathology*.

[121] Trautmann M., Menzel J., Bertling C. (2017). FUS-DDIT3 fusion protein-driven IGF-IR signaling is a therapeutic target in myxoid liposarcoma. *Clinical Cancer Research*.

[122] Miyama A., Kuratsu S., Takenaka S. (2019). Two case reports of intra-articular nodular fasciitis of the knee confirmed by MYH9-USP6 gene fusion expression. *Journal of Orthopaedic Science*.

[123] Endo M., Kohashi K., Yamamoto H. (2013). Ossifying fibromyxoid tumor presenting EP400-PHF1 fusion gene. *Human Pathology*.

[124] Antonescu C. R., Sung Y.-S., Chen C.-L. (2014). Novel ZC3H7B-BCOR, MEAF6-PHF1, and EPC1-PHF1 fusions in ossifying fibromyxoid tumors-molecular characterization shows genetic overlap with endometrial stromal sarcoma. *Genes, Chromosomes and Cancer*.

[125] Castro E., Cortes-Santiago N., Ferguson L. M. S., Rao P. H., Venkatramani R., López-Terrada D. (2016). Translocation t(7;12) as the sole chromosomal abnormality resulting in ACTB-GLI1 fusion in pediatric gastric pericytoma. *Human Pathology*.

[126] van IJzendoorn D. G. P., Sleijfer S., Gelderblom H. (2018). Telatinib is an effective targeted therapy for pseudomyogenic hemangioendothelioma. *Clinical Cancer Research*.

[127] Panagopoulos I., Gorunova L., Viset T., Heim S. (2016). Gene fusions AHRR-NCOA2, NCOA2-ETV4, ETV4-AHRR, P4HA2-TBCK, and TBCK-P4HA2 resulting from the translocations t(5;8;17)(p15;q13;q21) and t(4;5)(q24;q31) in a soft tissue angiofibroma. *Oncology Reports*.

[128] Davanzo B., Emerson R. E., Lisy M., Koniaris L. G., Kays J. K. (2018). Solitary fibrous tumor. *Translational Gastroenterology and Hepatology*.

[129] Mosquera J. M., Sboner A., Zhang L. (2013). Recurrent NCOA2 gene rearrangements in congenital/infantile spindle cell rhabdomyosarcoma. *Genes, Chromosomes and Cancer*.

[130] Alaggio R., Zhang L., Sung Y.-S. (2016). A molecular study of pediatric spindle and sclerosing rhabdomyosarcoma: identification of novel and recurrent VGLL2-related fusions in infantile cases. *The American Journal of Surgical Pathology*.

[131] Peng C., Guo W., Yang Y., Zhao H. (2008). Downregulation of SS18-SSX1 expression by small interfering RNA inhibits growth and induces apoptosis in human synovial sarcoma cell line HS-SY-II in vitro. *European Journal of Cancer Prevention*.

[132] El Beaino M., Araujo D. M., Lazar A. J., Lin P. P. (2017). Synovial sarcoma: advances in diagnosis and treatment identification of new biologic targets to improve multimodal therapy. *Annals of Surgical Oncology*.

[133] Möller E., Mandahl N., Mertens F., Panagopoulos I. (2008). Molecular identification of COL6A3-CSF1 fusion transcripts in tenosynovial giant cell tumors. *Genes, Chromosomes and Cancer*.

[134] Okimoto R. A., Wu W., Nanjo S. (2019). CIC-DUX4 oncoprotein drives sarcoma metastasis and tumorigenesis via distinct regulatory programs. *Journal of Clinical Investigation*.

[135] Kao Y.-C., Owosho A. A., Sung Y.-S. (2018). BCOR-CCNB3 fusion positive sarcomas: a clinicopathologic and molecular analysis of 36 cases with comparison to morphologic spectrum and clinical behavior of other round cell sarcomas. *The American Journal of Surgical Pathology*.

[136] Tran A., Tawbi H. A. (2012). A potential role for nilotinib in KIT-mutated melanoma. *Expert Opinion on Investigational Drugs*.

[137] Sleijfer S., Ray-Coquard I., Papai Z. (2009). Pazopanib, a multikinase angiogenesis inhibitor, in patients with relapsed or refractory advanced soft tissue sarcoma: a phase II study from the European organisation for research and treatment of cancer-soft tissue and bone sarcoma group (EORTC study 62043). *Journal of Clinical Oncology*.

[138] Wagner A. J., Kindler H., Gelderblom H. (2017). A phase II study of a human anti-PDGFR*α* monoclonal antibody (olaratumab, IMC-3G3) in previously treated patients with metastatic gastrointestinal stromal tumors. *Annals of Oncology*.

[139] Antonescu C. R., Besmer P., Guo T. (2005). Acquired resistance to imatinib in gastrointestinal stromal tumor occurs through secondary gene mutation. *Clinical Cancer Research*.

[140] Demetri G. D., van Oosterom A. T., Garrett C. R. (2006). Efficacy and safety of sunitinib in patients with advanced gastrointestinal stromal tumour after failure of imatinib: a randomised controlled trial. *The Lancet*.

[141] Demetri G. D., Reichardt P., Kang Y.-K. (2013). Efficacy and safety of regorafenib for advanced gastrointestinal stromal tumours after failure of imatinib and sunitinib (GRID): an international, multicentre, randomised, placebo-controlled, phase 3 trial. *The Lancet*.

[142] Chen L. L., Gouw L., Sabripour M., Hwu W.-J., Benjamin R. S. (2012). Combining targeted therapy with immunotherapy (interferon-*α*). *Oncoimmunology*.

[143] Falchook G. S., Trent J. C., Heinrich M. C. (2013). BRAF mutant gastrointestinal stromal tumor: first report of regression with BRAF inhibitor dabrafenib (GSK2118436) and whole exomic sequencing for analysis of acquired resistance. *Oncotarget*.

[144] Demicco E. G., Torres K. E., Ghadimi M. P. (2012). Involvement of the PI3K/Akt pathway in myxoid/round cell liposarcoma. *Modern Pathology*.

[145] Barretina J., Taylor B. S., Banerji S. (2010). Subtype-specific genomic alterations define new targets for soft-tissue sarcoma therapy. *Nature Genetics*.

[146] Kim A., Pratilas C. A. (2018). The promise of signal transduction in genetically driven sarcomas of the nerve. *Experimental Neurology*.

[147] Endo M., Yamamoto H., Setsu N. (2013). Prognostic significance of AKT/mTOR and MAPK pathways and antitumor effect of mTOR inhibitor in NF1-related and sporadic malignant peripheral nerve sheath tumors. *Clinical Cancer Research*.

[148] Tap W. D., Eilber F. C., Ginther C. (2011). Evaluation of well-differentiated/de-differentiated liposarcomas by high-resolution oligonucleotide array-based comparative genomic hybridization. *Genes, Chromosomes and Cancer*.

